# Atypical Presentation of Sphenoid Sinusitis With Prevertebral Involvement

**DOI:** 10.7759/cureus.67688

**Published:** 2024-08-24

**Authors:** Teng Huei Lee, Ramiza Ramza Ramli

**Affiliations:** 1 Department Otorhinolaryngology-Head and Neck Surgery, Hospital Universiti Sains Malaysia, Universiti Sains Malaysia, Kelantan, MYS

**Keywords:** headache, prevertebral abscess, nasopharyngeal abscess, sphenoid sinusitis, endoscopic sinus surgery (ess)

## Abstract

Isolated sphenoid sinusitis (ISS) is a rare but potentially serious condition, often leading to severe complications due to delayed diagnosis and treatment. This case report discusses a 75-year-old male with type 2 diabetes mellitus who presented with severe left-sided headache and neck pain. Diagnostic imaging revealed isolated sphenoid sinusitis with prevertebral extension, a rare occurrence that highlights the potential for deep neck space involvement. The patient underwent endoscopic transnasal incision and drainage of the prevertebral abscess with a left sphenoidotomy, resulting in full recovery without recurrence. This case emphasizes the importance of prompt recognition and intervention in ISS, particularly in cases with atypical presentations. The report also discusses the complex anatomy of the sphenoid sinus and its surrounding structures, the broad differential diagnosis of sphenoid sinus opacification, and the necessity for a multidisciplinary approach to management. This case contributes to the limited literature on ISS with prevertebral extension and underscores the critical need for early diagnosis and aggressive treatment to prevent severe complications.

## Introduction

Isolated sphenoid sinusitis (ISS) is a rare but clinically significant condition, representing only 1% to 2.7% of all sinus diseases [1]. This rarity, coupled with its nonspecific symptoms, often leads to delayed diagnosis and treatment, complicating clinical management [2]. Pathologies affecting the sphenoid sinus can lead to severe complications, including ocular diseases, cranial neuropathies, and intracranial conditions such as meningitis and brain abscesses [3]. The differential diagnosis for sphenoid sinus opacification is broad, encompassing inflammatory conditions, neoplasms, and benign cysts, with inflammatory lesions being the most frequent cause [4]. Understanding the anatomy of deep neck spaces is essential for recognizing the potential complications of sphenoiditis and its propensity to spread. The fascial planes create potential spaces that facilitate the rapid dissemination of infections, including the retropharyngeal, danger, and prevertebral spaces [5]. The prevertebral space, in particular, is significant due to its potential involvement in severe infections and abscess formation [6].

In this specific case, the patient presented with symptoms of a left-sided headache and neck pain but no neurological deficits, which is notable given the extension of the infection from the sphenoid sinus into the prevertebral space. Such an extension is poorly documented in the literature, making this an unusual and complex presentation [7]. The rarity of this condition and its complex anatomical involvement underscore the importance of an accurate and timely diagnosis. Effective management of ISS often necessitates a combination of surgical and medical interventions. Endoscopic sinus surgery is commonly the preferred treatment modality, allowing for comprehensive drainage and exploration of the sphenoid sinus and any associated collections [8]. This surgical approach not only facilitates the removal of the infected material but also enables the collection of specimens for microbiological analysis, thereby enhancing the efficacy of targeted antimicrobial therapy [9].

## Case presentation

A 75-year-old gentleman with underlying type 2 diabetes mellitus presented to the emergency department in a rural community hospital complaining of a severe headache for two days. The pain was excruciating and throbbing in nature and felt more over the left side of the head, radiating down to the left side of the neck. His sleep was disturbed and not relieved with over-the-counter (OTC) analgesics, prompting him to seek treatment at the hospital. A week prior to the current symptoms, the patient claimed to have a history of upper respiratory tract infection, i.e., fever, mild cough, and sore throat. Otherwise, he denied any photophobia, neurological, rhinitis, otological, or odontological symptoms.

**Figure 1:**
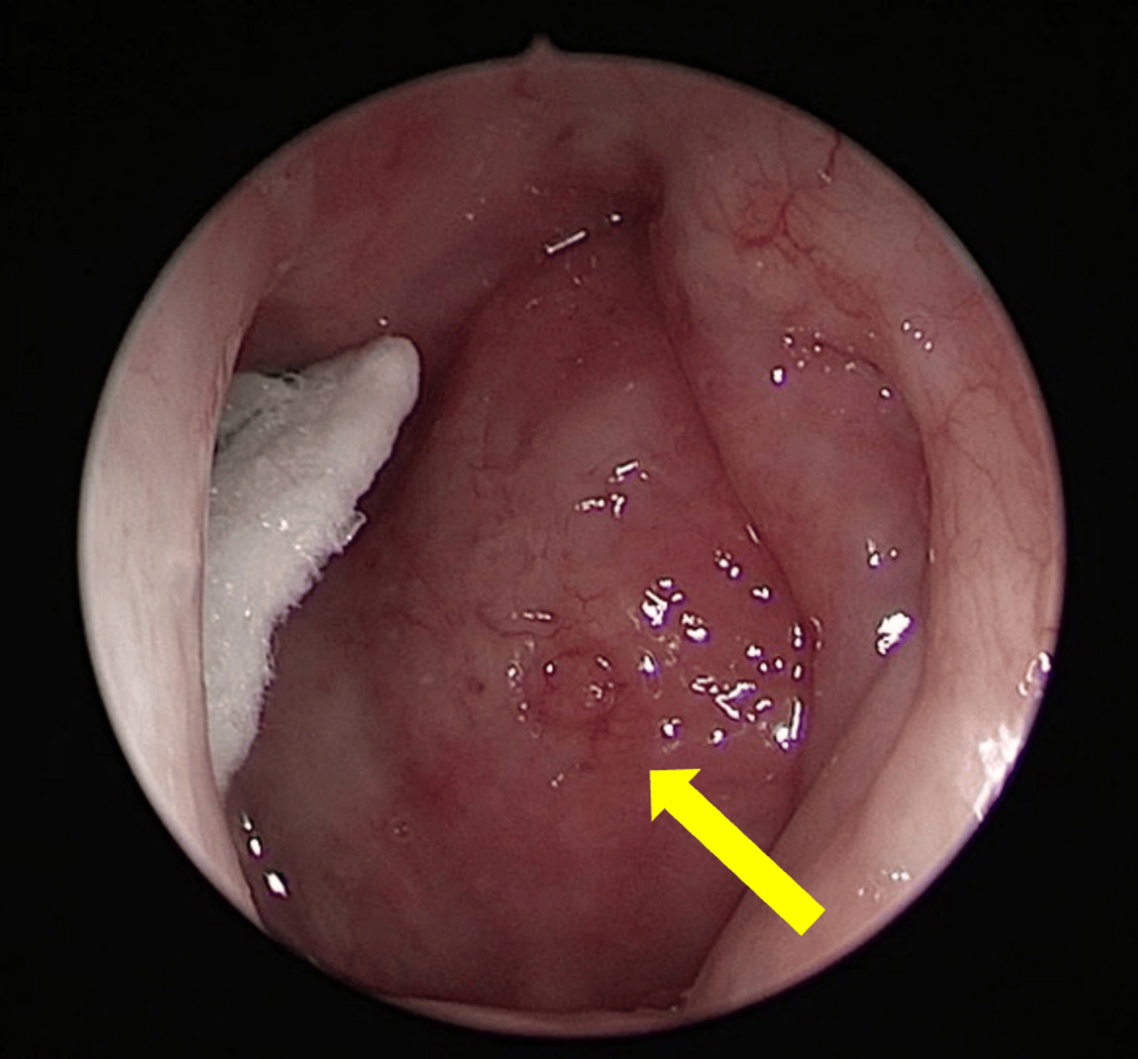
Nasoendoscopic view of the left side of the nasopharynx The yellow arrow indicates the area of the nasopharyngeal bulge.

On examination, he was alert and conscious with a Glasgow Coma Scale (GCS) of 15 but appeared to be anxiously in pain. His vital signs revealed elevated blood pressure of 156/100 mmHg and a pain score of eight out of 10, while other signs were unremarkable. His pupils’ size was 2 mm bilateral and reactive to light; all limbs were mobile with good tone and power; and neurological reflexes were normal. On rigid nasal endoscopy, the left nasopharyngeal wall was seen bulging and inflamed, filling up the left fossa of Rosenmüller (FOR) (Figure 1). There were no neck nodes palpable, and his intraoral examination was normal.

**Figure 2:**
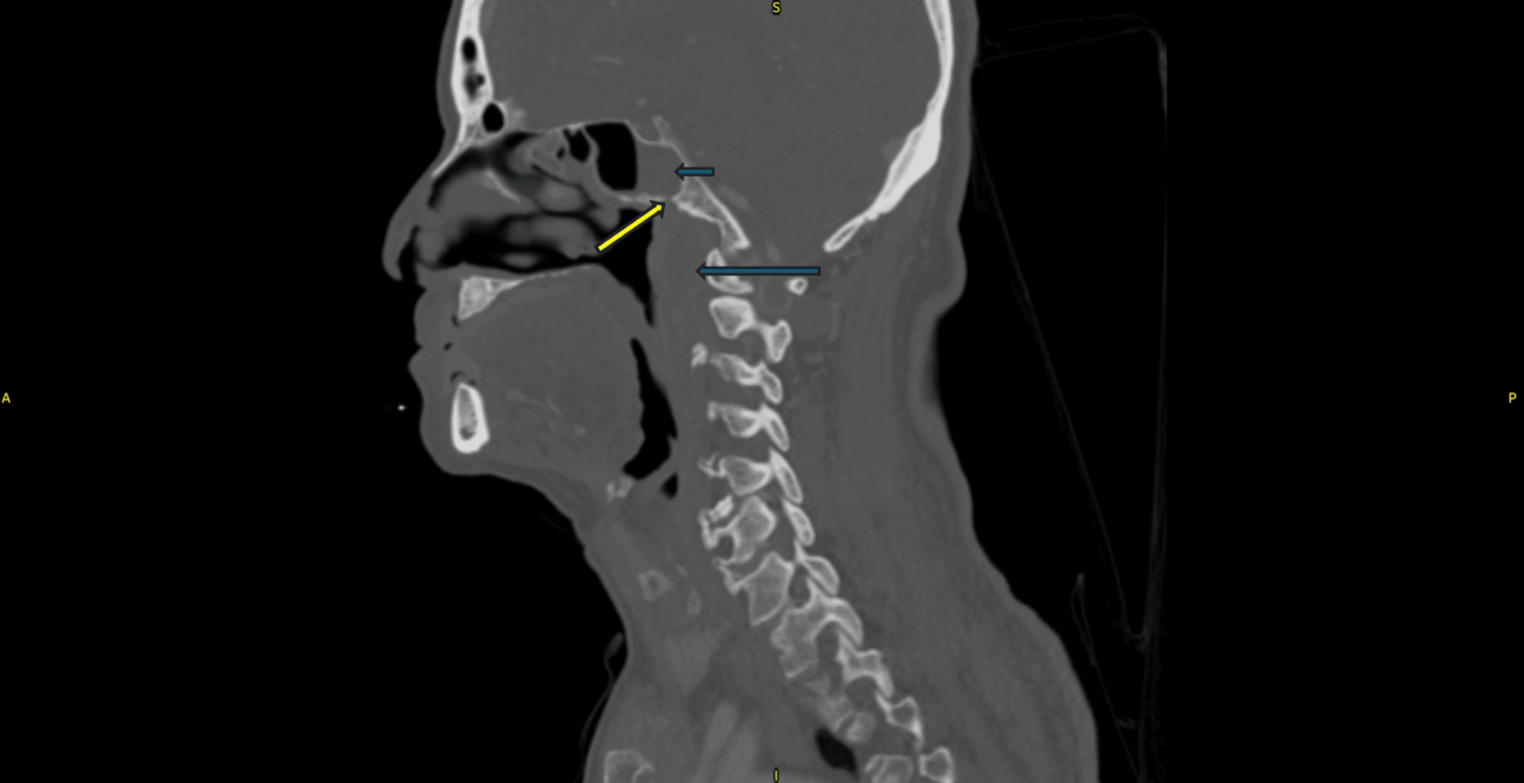
Contrast-enhanced CT (bone window) scan in sagittal view Isolated sphenoid collection in the sphenoid sinus (short arrow), thickened prevertebral space at the nasopharyngeal region (long arrow), and bony lytic erosion at the floor of the sphenoid sinus (yellow arrow) are observed.

A contrast-enhanced computer tomography (CECT) of the paranasal sinuses showed left prevertebral intramuscular collection with retropharyngeal collection measuring 2 x 2.9 x 3.5 cm from the clivus superiorly and extending inferiorly to C2-3 vertebrae. There was an isolated opacification in the left sphenoid sinus with bony lytic erosion at the floor of the sphenoid (Figure 2). Blood investigations revealed leucocytosis, with elevated erythrocyte sedimentation rate (ESR) and C-reactive protein (CRP) (Table 1). 

**Table 1:** Serial laboratory investigations

Laboratory investigations	Normal range	Before the surgical intervention	After the surgical intervention	One month after the surgical intervention
Full blood count (FBC)				
Hemoglobin (g/dL)	13.8–17.2	13.4	13.8	13.9
Total white cell count (x10^9^ /L)	4.0-11.0	16.9	12.3	6.4
Platelet (x10^9^ /L)	150-400	236	320	250
Inflammatory markers				
Erythrocyte sedimentation rate (mm)	Less than 15	44	23	10
C-reactive protein (mg/L)	0.8-1.0	92	3.1	0.9

Endoscopic transnasal incision and drainage of the prevertebral abscess and left sphenoidotomy were performed under general anesthesia. Intraoperatively, we yielded 10 ccs of thick yellowish purulent discharge from the prevertebral space, with the underlying prevertebral muscle appearing bulky and unhealthy with sloughy tissue seen overlying it. The left sphenoid sinus was filled with 5 ccs of thick yellowish purulent discharge, and the underlying sinus mucosa appeared inflamed. Postoperatively, he was administered intravenous (IV) co-amoxiclavulanic acid 1,200 milligrams (mg) for one week and oral paracetamol 1,000 mg six hourly for pain control. Cultures for tuberculosis were negative; pus bacterial and tissue bacterial cultures grew *Klebsiella pneumoniae*; fungal cultures revealed no growth. Tissue sent for histopathological examination (HPE) from the left FOR and deep prevertebral muscle showed acute inflammatory changes with no malignant features seen. His blood parameters also showed improved leucocytosis and ESR and CRP levels. 

**Figure 3:**
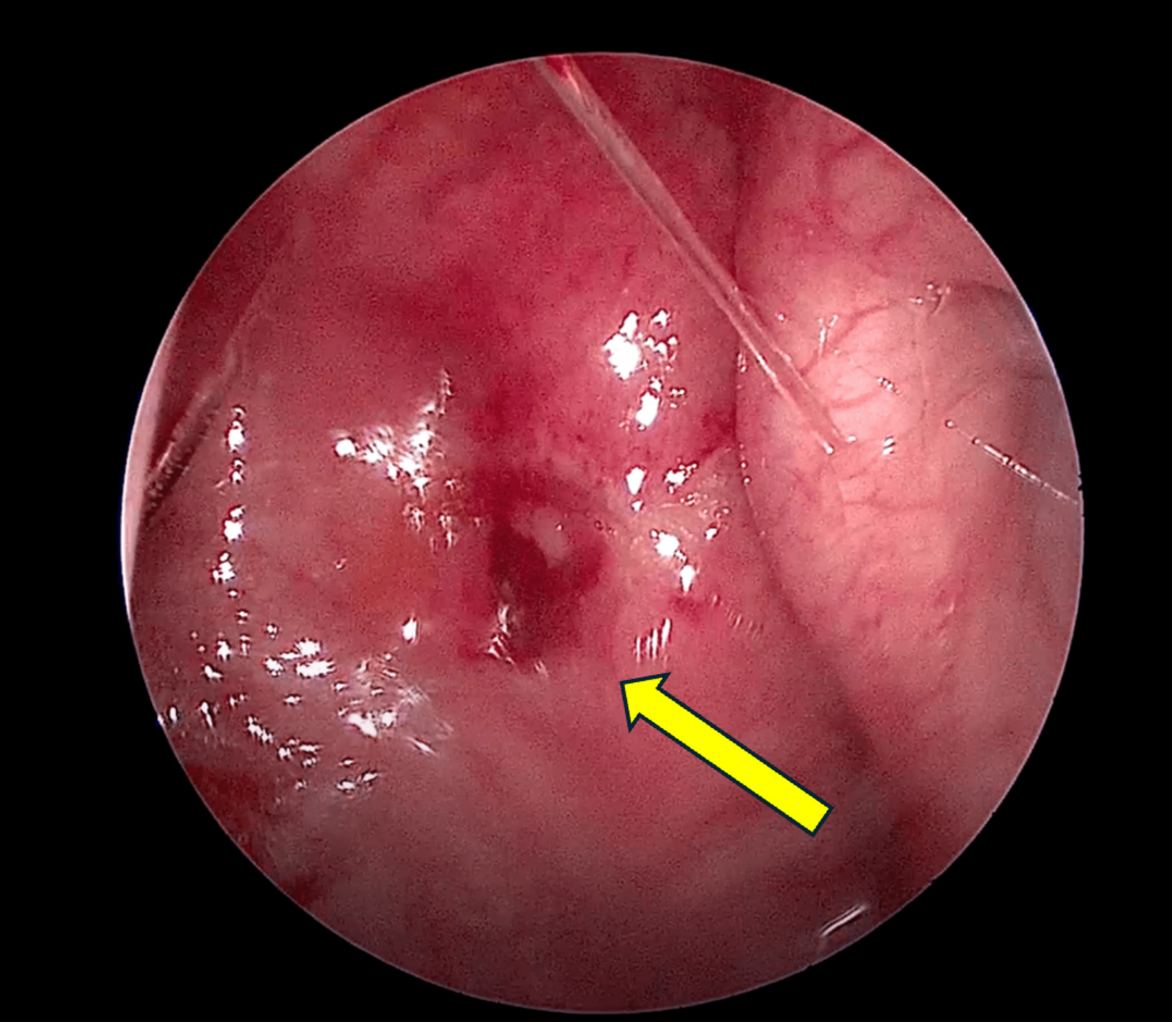
The nasoendoscopic view of the left side of the nasopharynx reveals a resolved nasopharyngeal bulge. Site of incision and drainage (yellow arrow)

**Figure 4:**
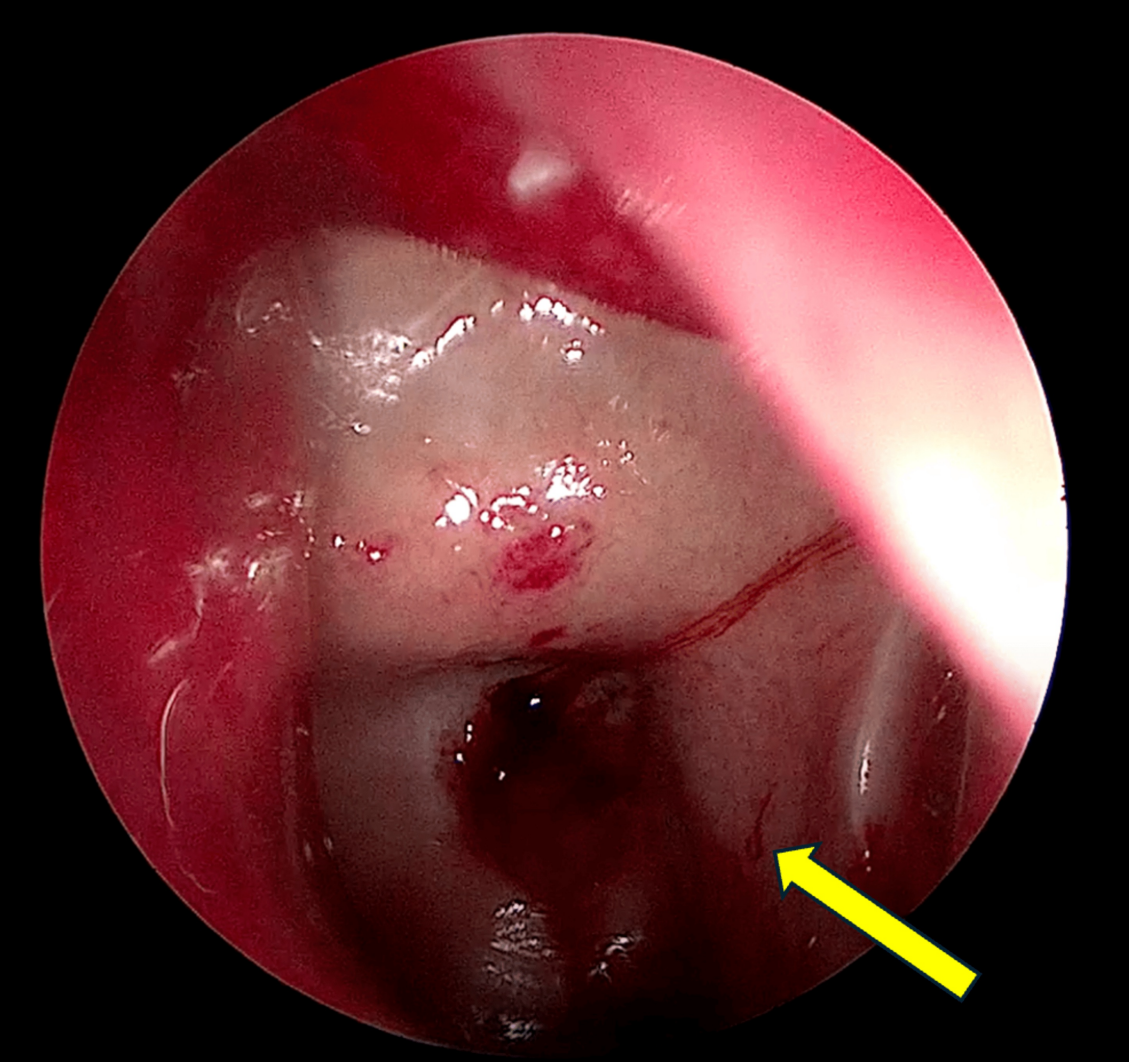
The nasoendoscopic view of the left sphenoid sinus reveals a patent, widened left spenoid ostia. Healthy sphenoid sinus mucosa (yellow arrow)

He made a full recovery after the surgery and completed oral co-amoxiclavulanic acid (625 mg, every eight hours) for one week with regular isotonic alkaline nasal douching. There were no recurring headaches or neck pains upon subsequent outpatient clinic follow-up. The outpatient nasal endoscopic assessment revealed a resolved nasopharyngeal bulge (Figure 3) and a patent left sphenoid ostium (Figure 4).

## Discussion

The sphenoid sinus, located within the sphenoid bone, is anatomically situated below the sella turcica, which houses the pituitary gland and is bordered laterally by the cavernous sinuses. This anatomical relationship places the sphenoid sinus in close proximity to several critical structures, including the optic nerves, carotid arteries, oculomotor nerve, abducens nerve, trochlear nerve, and branches V1 and V2 of the trigeminal nerve. This proximity makes diseases of the sphenoid sinus clinically significant, as they can result in severe complications such as ocular diseases, cranial neuropathies, and intracranial complications like meningitis and brain abscesses [1].

Sphenoiditis often originates from inflammation spreading from the posterior ethmoid sinuses. It is referred to as isolated sphenoiditis when other sinuses are unaffected. Isolated sphenoid sinus disease is relatively rare, affecting only 1% to 2.7% of patients diagnosed with paranasal sinus disease [2]. The differential diagnosis for sphenoid sinus opacification includes a wide range of conditions, such as inflammatory diseases, neoplasms, and benign cysts. Neoplasms, though rare, typically arise from invasion by adjacent skull base structures [3]. In 50.3% of isolated sphenoiditis cases, the causes are inflammatory, 20.2% are mucoceles, and the remainder include cerebrospinal fluid (CSF) leaks, encephaloceles, fibrous dysplasia, inverted papillomas, and malignant tumors [3].

Gaining insight into the anatomy of deep neck areas is essential for understanding the complications and potential spread of sphenoiditis to the prevertebral space, as seen in this case. The fascial planes create potential spaces that facilitate the rapid spread of infections. The retropharyngeal space lies immediately behind the pharynx, followed by the danger space, and then the prevertebral space, separated by the alar and prevertebral fascia, respectively [4]. As the prevertebral fascia runs superiorly from the base of the clivus to the coccyx, bone erosions of the sphenoid floor, as observed in this instance, offer an anatomically reasonable pathway for dissemination. However, it is uncommon for a deep neck infection to spread from the base of the skull; therefore, this case of sphenoiditis involving the longus colli muscle and extending into the prevertebral region is rare and unusual [5].

The microbiology of prevertebral infections is usually polymicrobial, necessitating broad-spectrum empiric antibiotic coverage until culture results are available. It is important to isolate these organisms for optimal antimicrobial therapy; however, the inability to isolate these organisms is not uncommon [6]. *Staphylococci *are the most commonly cultured pathogens, but other pathogens include *Streptococcus pneumoniae*, aerobic Gram-negative bacilli such as *Pseudomonas aeruginosa*, *Klebsiella pneumoniae*, *Haemophilus influenzae*, *Escherichia coli*, and anaerobes including *Peptostreptococcus*, *Fusobacterium*, *Prevotella*, and *Porphyromonas *[7].

Proper evaluation of sinusitis with suspected posterior extension requires radiological imaging to look for the formation of abscesses. Given that more than 75% of prevertebral abscesses do not present with neck or back pain, CECT is the gold standard for diagnosing deep neck infections [8]. A CECT shows rim enhancement of the abscess, mucosal and bony paranasal sinus inflammation, and bony erosion. In contrast, magnetic resonance imaging (MRI) is advantageous for detailed soft tissue definition and differentiating inspissated mucus from intra-sinus masses [7,9].

The primary strategy for managing sphenoiditis with prevertebral extension involves prompt surgical drainage of the infected collection in conjunction with antimicrobial therapy. Surgical drainage is imperative, as it allows for the precise identification of the causative pathogens, thereby facilitating targeted antimicrobial treatment. Evidence from various studies indicates that conservative management using antimicrobial therapy alone often results in suboptimal outcomes compared to surgical intervention [5,8]. In this case, endoscopic sinus surgery was employed as the treatment modality. This method was chosen because it provided comprehensive drainage and exploration of the infection within the left sphenoid sinus and the prevertebral collection localized to the nasopharyngeal region. The endoscopic approach not only ensured the effective removal of the infected material but also allowed for the collection of specimens for microbiological analysis, thereby enhancing the efficacy of the targeted antimicrobial therapy.

## Conclusions

This case report highlights the successful management of a rare presentation of ISS with prevertebral extension. The key findings emphasize the importance of early and accurate diagnosis in preventing serious complications associated with deep neck space infections. The prompt surgical intervention, combined with targeted antibiotic therapy, led to a favorable outcome in this patient. The case underscores the necessity for clinicians to maintain a high index of suspicion for ISS in patients with atypical headaches and neck pain, especially in those with predisposing factors such as diabetes mellitus. The implications of this report suggest that endoscopic surgery remains the preferred treatment modality in such complex cases, ensuring both effective drainage and specimen collection for precise microbiological diagnosis. The report adds to the growing body of evidence supporting early surgical intervention as a critical component in the successful treatment of isolated sphenoiditis with prevertebral involvement.
